# Efficacy and safety of acupuncture treatment for fatigue after COVID-19 infection: study protocol for a pilot randomized sham-controlled trial

**DOI:** 10.3389/fneur.2023.1302793

**Published:** 2023-11-15

**Authors:** Sung-A. Kim, Ji-Su Lee, Taegon Kim, Tae-Hun Kim, Sunoh Kwon, Jung Won Kang

**Affiliations:** ^1^Department of Clinical Korean Medicine, Graduate School, Kyung Hee University, Seoul, Republic of Korea; ^2^Korean Medicine Clinical Trial Center, College of Korean Medicine, Kyung Hee University, Seoul, Republic of Korea; ^3^Korean Medicine Convergence Research Division, Korea Institute of Oriental Medicine, Daejeon, Republic of Korea; ^4^Department of Acupuncture & Moxibustion, College of Korean Medicine, Kyung Hee University, Seoul, Republic of Korea; ^5^Department of Acupuncture and Moxibustion, Kyung Hee University Medical Center, Seoul, Republic of Korea

**Keywords:** Long COVID, fatigue, acupuncture, randomized controlled trial, sham acupuncture

## Abstract

**Background:**

As the coronavirus disease 2019 (COVID-19) pandemic has spread globally, its sequelae, called Long COVID, have persisted, troubling patients worldwide. Although fatigue is known to be the most frequent among Long COVID symptoms, its mechanism and treatment have not been clearly demonstrated. In 2022, we conducted a preliminary prospective case series and found that acupuncture and moxibustion were feasible interventions for fatigue. This study is a pilot patient-assessor-blinded randomized sham-controlled trial to evaluate the efficacy and safety of acupuncture treatment for patients with fatigue that has persisted for at least 4 weeks after recovery from COVID-19.

**Methods:**

Thirty patients will be recruited and randomly assigned to either the acupuncture or sham acupuncture treatment groups. Treatment will be conducted thrice a week for both groups during 4 weeks. The primary outcome will be the efficacy and safety of acupuncture, including numeric rating scale (NRS), brief fatigue inventory (BFI), fatigue severity scale (FSS), and adverse event evaluation. Secondary outcomes will be evaluation of improvement in the comorbid symptoms of fatigue and feasibility variables. Outcome variables will be assessed before treatment, 4 weeks after treatment, and 8 weeks after treatment completion.

**Discussion:**

The results of this study will be used to clarify the efficacy and safety of acupuncture treatment for persistent fatigue in patients with Long COVID. Additionally, the feasibility of the study design was validated to provide evidence for future full-scale randomized controlled trials.

**Clinical trial registration**: identifier: KCT0008656 https://cris.nih.go.kr/cris/search/detailSearch.do?seq=24785&search_page=L.

## Introduction

Long COVID manifests as a wide range of newly developed symptoms that persist after initial acute SARS-CoV-2 infection (1). While the COVID-19 pandemic has lasted for more than 2 years, most symptoms of the acute phase have become mild or even asymptomatic, but its long-term consequences persist for months or longer (2). Although survivors must return to society after recovering from COVID-19, their ongoing health problems impair their quality of life and ability to work in the absence of standardized treatment. These prolonged implications remain a global public health burden rather than COVID-19 itself (3).

Among more than fifty known post-COVID conditions, fatigue has been reported as the most common symptom, affecting 58% of patients with Long COVID (4). Long COVID fatigue is defined as the decrease in physical and/or mental performance that results from changes in central, psychological, and/or peripheral factors, which is different from objective fatigability (5). It occurs more in women significantly and is usually accompanied with fatigue-related symptoms such as stress, anxiety, depression and cognitive impairment (6, 7). Its pathophysiology remains unknown, and several clinical recommendations suggest temporary self-management and multidisciplinary rehabilitation for its treatment (8). Although fatigue symptoms reduce daily activity, motivation, and concentration, the fact that patients must vaguely endure fatigue without reliable treatment interventions has increased their anxiety. Therefore, complementary and alternative medicine (CAM) interventions have been sought as treatment and management strategies (9).

Acupuncture is a potential intervention for fatigue in patients with Long COVID (10). Acupuncture has been demonstrated to be an effective and safe modality for cancer-related fatigue and chronic fatigue syndrome by alleviating neuroinflammatory response, modulating immune responses, regulating neuropeptides (e.g., dopamine, *β*-endorphin), promoting oligodendrocyte proliferation and stimulating NO production (11–13). Although it has been used practically for fatigue symptoms in other diseases, only a few case studies have explored the possible application of acupuncture treatment for persistent fatigue following COVID-19 (14).

In 2022, our research team will complete a preliminary case series study to assess the feasibility of acupuncture and moxibustion treatment for several Long COVID symptoms. In this case series, all 16 participants complained of multiple symptoms, such as fatigue, olfactory disturbance, joint pain, and alopecia after COVID-19 infection. Because fatigue was assessed as the most severe symptom, the subjects expected to be treated mostly for this. The participants ended up seeking CAM interventions, such as acupuncture or moxibustion treatment, as the specific cause could not be determined even after testing at a tertiary general hospital, and there was no dependable treatment for postviral fatigue. After 12 treatment sessions, we confirmed a significant improvement in fatigue, which persisted even after 4 weeks of follow-up evaluation. In addition, there were no severe adverse events, except for a few mild blisters after moxibustion treatment.

To our knowledge, no randomized sham-controlled trial (RCT) has evaluated the efficacy and safety of acupuncture for treating fatigue after COVID-19. Therefore, we designed a pilot RCT based on our previous prospective case study to assess the efficacy and safety of acupuncture for fatigue after COVID-19 infection compared with a control group treated with non-penetrating sham acupuncture because of its physiologically inert characteristics.

## Study objectives

The aims of this pilot RCT will be as follows: (1) to test the efficacy and safety of acupuncture treatment for patients experiencing persistent fatigue after recovery from COVID-19 and (2) to explore the feasibility of future full-scale RCT.

## Materials and methods

This protocol followed the Standard Protocol Items: Recommendations for Interventional Trials (SPIRIT) (Supplement 1) (15). The trial was listed on the Clinical Research Information Service (CRIS) registry with study ID KCT0008656 in July 2023.

### Trial design and setting

**Figure 1:**
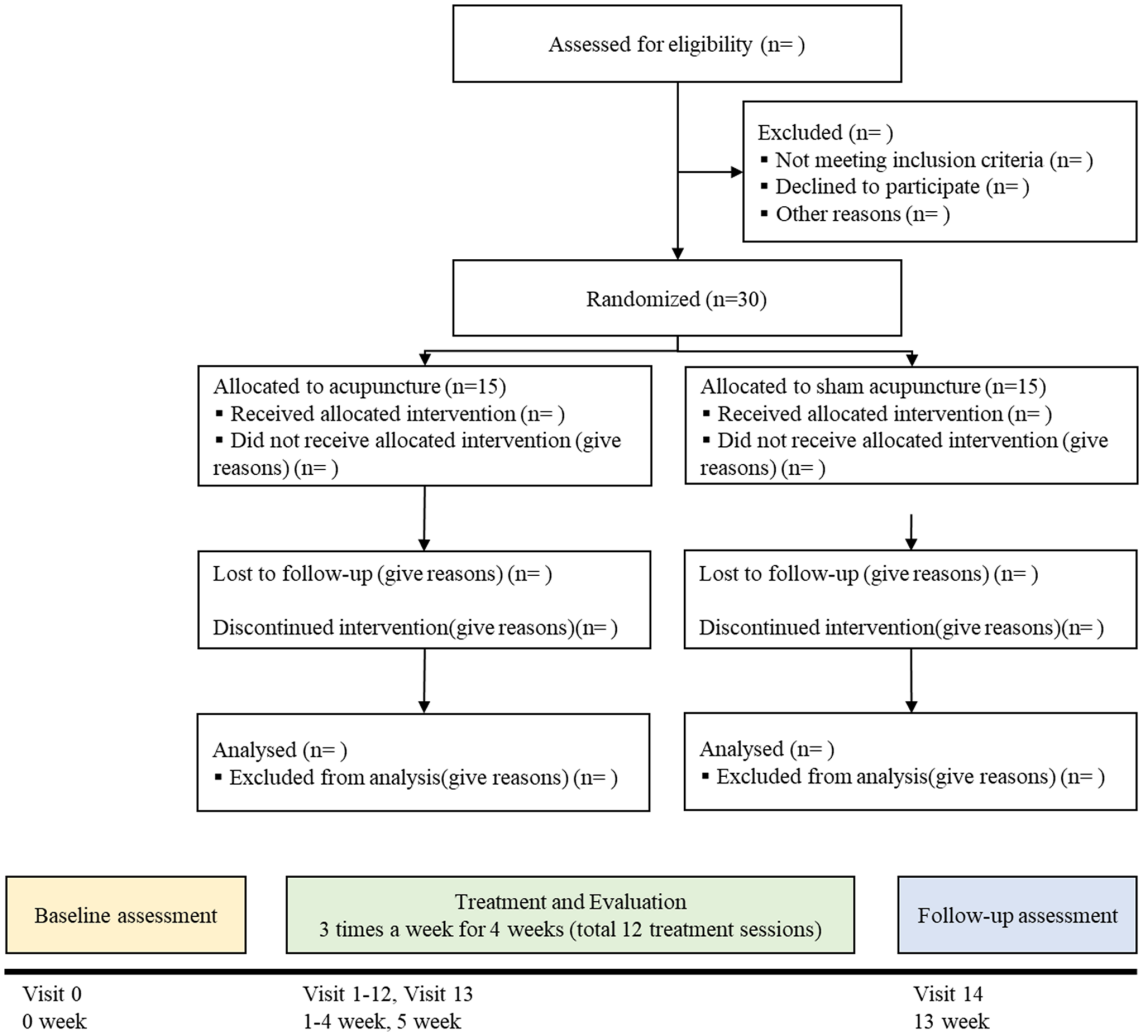
Flow chart of study.

**Figure 2:**
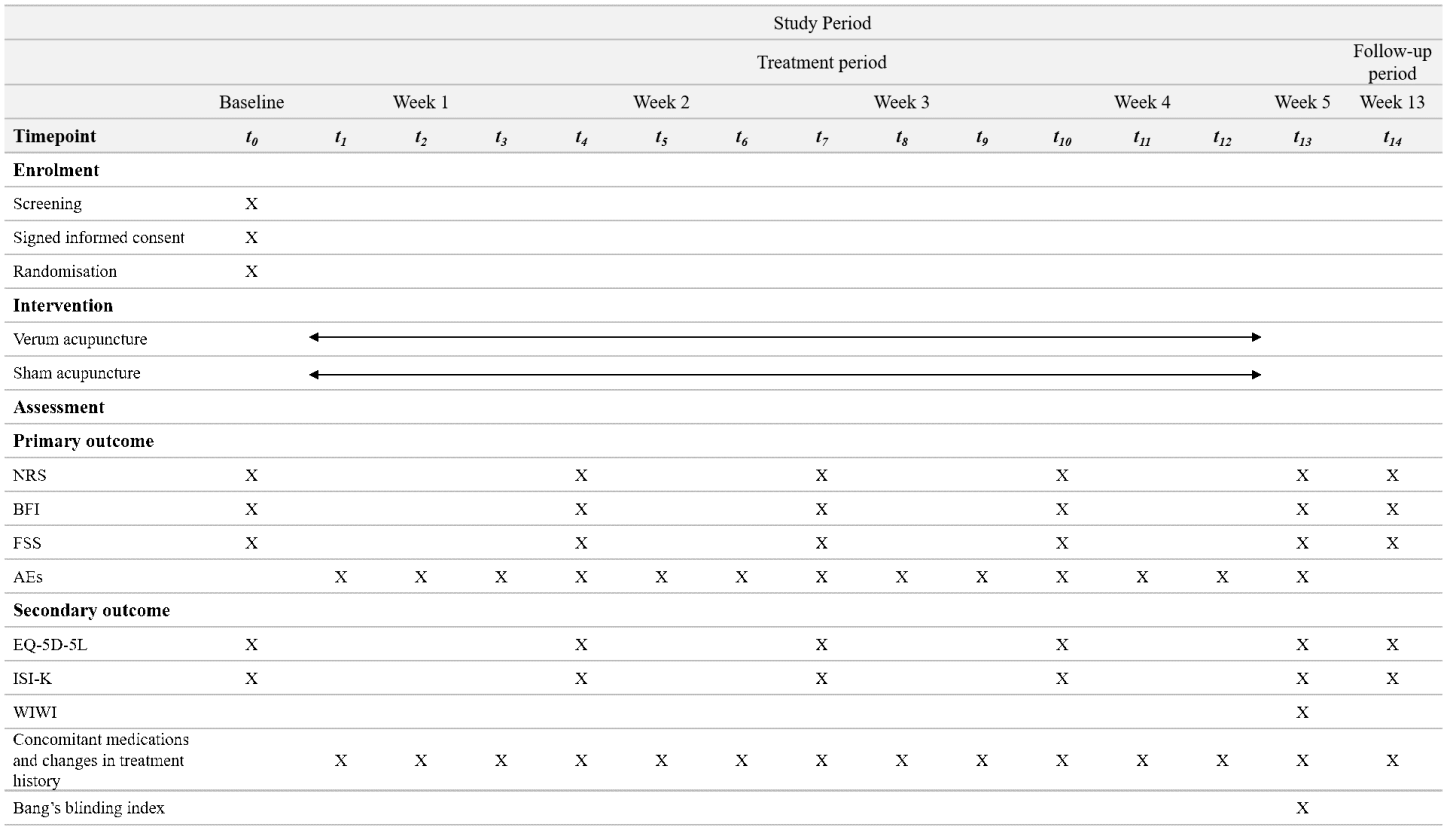
SPIRIT schedule of enrollment, interventions, and assessments. *SPIRIT, standard protocol items: recommendations for interventional trials; NRS, numeric rating scale; BFI, brief fatigue inventory; FSS, fatigue severity scale; AEs, adverse events; EQ-5D-5L, euroQol-5-dimension 5 level; ISI-K, korean-validated version of the insomnia severity index; WIWI, Was It Worth It questionnaire.

A two-arm, randomized, patient-assessor-blinded, sham-controlled trial will be conducted at the Kyung Hee University Korean Medicine Hospital in Seoul, Korea. This study was approved by the Institutional Review Board (IRB) of Kyung Hee University Korean Medicine Hospital (KOMCIRB 2023-04-006-001). Thirty participants will be recruited through subway advertisements and posters. Patient enrollment will begin in August 2023 and is expected to end in November 2023. Eligible and consenting patients will be randomly allocated to either the verum acupuncture or the sham acupuncture group at a ratio of 1:1. All subjects will receive twelve sessions of treatment for 4 weeks and will be assessed at baseline, week 4, and week 12. The trial design is summarized in Figure 1. Figure 2 outlines the SPIRIT schedule for enrollment, interventions, and assessments.

### Participants

A formal sample size calculation was not performed because this was designed as a pilot study to calculate the appropriate sample size for subsequent RCTs. Our study will recruit 30 patients, based on previous studies that recommended the minimum number of people necessary for preliminary research in the field of life sciences to be 10 to 35, considering a 20% drop-out (16–18).

Participants who have the following conditions will be included: (1) aged 20 to 65 years; (2) diagnosed with COVID-19 and quarantined (and cured) at least 1 month ago; (3) experiencing newly developed fatigue that has persisted for more than 4 weeks after being cured of COVID-19, and whose symptom intensity is 4 or higher on the 0–10 numeric rating scale (NRS); and (4) voluntarily agreeing to participate in the study through a written consent form after listening to the purpose and characteristics of this clinical study.

Participants who have one or more of the following conditions will be excluded: (1) having history of diseases (cancer, anemia, hypothyroidism or psychiatric disease such as depression) or surgical operation that can cause fatigue symptom before the infection of COVID-19; (2) having fear of acupuncture treatment; (3) pregnant, breast feeding or expecting a pregnancy during the study period; (4) judged to find it difficult to comply with this treatment protocol (e.g., visiting the hospital, or filling in a questionnaire); (5) participating in another clinical study; (6) with other factors deeming them inadequate for participation in the view of research investigators.

Eligible patients will be informed of the aims and potential benefits, risks, alternatives, and responsibilities of the study by a Korean Medicine (KM) doctor throughout the consent process and written consent will be obtained. The patients’ information will remain confidential, and they will have the right to withdraw without prejudice at any time.

### Randomization and blinding

Randomization will be performed by an independent statistician using a computerized randomization with R package (R Version 4.3.1, Windows) with 1:1 allocation ratio. Prior to randomization, the study coordinator will ensure that each participant met the inclusion and exclusion criteria and informed consent is obtained. Random allocation will be conducted by an independent study coordinator, who will open the corresponding envelope in front of the participant.

The participants, outcome assessors, and statisticians will be blinded to the allocation, whereas practitioners will not. To evaluate the blinding, patients will assess their allocation guess and credibility rating immediately after the last treatment session.

### Intervention

Reporting of intervention in this protocol followed the Standards for Reporting Interventions in Clinical Trials of Acupuncture (STRICTA) guidelines (Supplement 2) (19). Acupuncture treatment will be performed by certified practitioners with at least 6 years of KM education and more than 2 years of clinical experience. Regardless of their allocation, all participants will be treated for a total of 12 sessions (three times per week for 4 weeks) in a similar environment to a KM hospital setting with a blinding curtain (Supplement 3). The standard operating procedure (SOP) will be developed by the principal investigator and co-investigators, and acupuncture practitioners will be required to attend a training course on standardized treatment before the beginning of the study.

### Verum acupuncture treatment

Fixed acupuncture will be provided to patients in the verum acupuncture treatment group. The acupuncture points were selected according to the consensus of board-certified professional KM doctors based on a textbook, literature review, and the points used in our previous prospective case series study (20–22). Ten acupuncture points were chosen: CV12, CV4, bilateral LI4, SP6, ST36, and LI11, in accordance with the WHO Standard Acupuncture Point Locations (Table 1).

**Table 1:** Acupuncture point locations.

Acupuncture point	Location
CV12	On the upper abdomen, 4 cun superior to the center of the umbilicus, on the anterior median line.
CV4	On the lower abdomen, 3 cun inferior to the center of the umbilicus, on the anterior median line.
LI4	On the dorsum of the hand, radial to the midpoint of the second metacarpal bone.
SP6	On the tibial aspect of the leg, posterior to the medial border of the tibia, 3 cun superior to the prominence of the medial malleolus.
ST36	On the anterior aspect of the leg, on the line connecting ST35 with ST41, 3 cun inferior to ST35.
LI11	On the lateral aspect of the elbow, at the midpoint of the line connecting LU5 with the lateral epicondyle of the humerus.

For patient blinding, the Park Sham Acupuncture Device (Dong Bang Acuprime, United Kingdom) will be attached to the acupuncture points using double-sided tape. Sterilized disposable 25 mm × 40 mm acupuncture needles (Dong Bang, South Korea) will be used at a depth of 5–20 mm, depending on the anatomical location of the acupuncture point. After insertion, needles will be twisted several times until the participants feel “de qi” sensation and be left for 20 min. Infrared therapy will be administered to the abdomen during needle retention.

### Sham acupuncture treatment group

For the sham acupuncture treatment group, 25 mm × 45 mm-size non-penetrating needles devised by Park et al. (Acuprime, United Kingdom) will be used (23, 24). These are sterilized disposable blunt needles that only contact the skin without penetration and will be propped by a validated guiding tube called the Park Sham Acupuncture Device (Dong Bang Acuprime, UK) which will be attached to the same acupuncture points as those of the verum acupuncture group. There will be no manipulation of qi, and the needles will be retained for 20 min.

### Outcomes

Outcomes will be assessed by independent KM doctors who will be blinded to the randomization. The outcome assessors will be trained before the study begins and will follow the SOP of this trial.

### Primary outcome

The primary outcomes are: (1) NRS of fatigue, (2) brief fatigue inventory (BFI), (3) fatigue severity scale (FSS), and (4) evaluation of adverse events (AEs). Outcome measures will be collected at baseline and at weeks 5 and 13.

The NRS for fatigue is a self-assessment of the average intensity of fatigue symptoms during the past week. Each participant will score on a 0–10 scale.

The BFI consists of nine items using numeric scales from 0 to 10 to assess the severity and interruption of daily life during the previous 24 h. Three items evaluate the degree of current fatigue from the usual to the worst level, while the other six items assess disturbances in daily activity. The total BFI score is calculated as the mean of the nine-item scores. A previously validated Korean version of the BFI will be used (25).

The FSS contains nine statements that evaluate the severity of fatigue symptoms using a 7-point Likert self-rating scale. The score will be calculated as the sum of the nine item scores, and the cut-off value is thirty-six. A previously validated Korean version of the FSS will be used (26).

AEs related to acupuncture treatment include needle-site pain, skin bruising, bleeding, dizziness, anxiety, and infection. All unexpected and unintended responses related to acupuncture will be recorded by the investigators after each treatment session. A causal relationship between treatment and AEs will be categorized using a 1–6 scale (1 = definitely related; 2 = probably related; 3 = possibly related; 4 = probably not related; 5 = definitely not related; 6 = unknown), and the severity of AEs will be scored using Spilker’s classification (1 = mild; 2 = moderate; 3 = severe).

### Secondary outcome

In addition to the primary outcome measurements, secondary outcomes will assess the comorbid symptoms of fatigue and feasibility variables of this study.

EuroQol-5-dimension 5 level (EQ-5D-5L) consists of 5 dimensions: mobility, self-care, usual activities, pain/discomfort, and anxiety/depression. It is used to assess overall quality of life by selecting the most appropriate statement among each of the five dimensions. The EQ-5D-5L will be administered at baseline, week 5, and week 13.

The Korean-validated version of the Insomnia Severity Index (ISI-K) assesses insomnia severity. It consists of 7 items using a 0–4 Likert scale. The sum of these scores becomes the total score, ranging from 0 to 28, and a cutoff score for discriminating insomnia patients is considered 15.5 (27). The ISI-K score will be assessed at baseline and at weeks 5 and 13.

Concomitant medications and changes in the treatment history will also be investigated. As all pharmacological or CAM interventions for any reasons will be not prohibited during the trial period, it will be recorded narratively at every visits.

The Was It Worth It (WIWI) questionnaire is to assess the feasibility of a clinical study by gathering participants’ opinion about their experiences in the study. It consists of 5 items, and subjects can choose one of 3 narrative answers. The results are evaluated qualitatively, not quantitatively (28). The WIWI will be measured at week 5.

For successful patient blinding, all subjects will be asked to guess whether they are allocated to the verum or sham acupuncture group at week 5. According to the correctness of the answer, it would be coded −1 or 1. If they answer that they do not know, it will be coded as 0.

To investigate the feasibility of the pilot RCT, the appropriateness of the study design, such as subject compliance to treatment and outcome sessions, selection rate during the study period, reasons for exclusion, and dropout rate, will be analyzed.

### Statistical analysis

Statistical analyses will be performed on both an intention-to-treat (ITT) basis and per-protocol (PP) population (95% confidence interval [CI]) using the SAS package program (Version 9.4; SAS Institute Inc., United States). Missing data will be replaced according to the last observation carried forward (LOCF) method. Baseline characteristics will be shown as mean ± standard deviation (SD) for continuous data and n (%) for categorical data. To analyze the baseline characteristics, we will conduct between-group comparisons at baseline using two-sample *t*-test or Wilcoxon rank sum test for continuous data and chi-squared test or Fisher’s exact test for categorical data with *p* < 0.05, as statistically significant, to determine whether the data are normally distributed.

For primary and secondary outcome measures, the mean difference from the baseline values to the end of treatment in each group will be compared using a two-sample *t*-test or Wilcoxon rank-sum test. If we needed to compare repeated measures, we would implement the repeated measures ANOVA. If any imbalances in baseline characteristics between groups were encountered, we will conduct analysis of covariance (ANCOVA) using these variables as covariates and the randomized group as a fixed factor. We might conduct subgroup analysis according to the baseline demographics, such as analysis on symptom duration over or under than 3-month period. In addition, to compare pre and post treatment assessment, we would use paired *t*-test, after evaluating the normality of the data. The safety and feasibility variables are descriptively presented with detailed explanations. Blinding assessment will be analyzed using Bang’s blinding index (29).

### Ethics and dissemination

The study protocol was approved by the IRB of Kyung Hee University Korean Medicine Hospital (KOMCIRB 2023–04–006-001). Informed consent will be obtained from all the participants. Participation in this study is completely voluntary, and participants are able to withdraw their participation at any time, if desired, without any consequences related to their treatment or follow-up. If control group participants desire to receive the verum treatment after the completion of study, we will treat them in our outpatient clinic. In addition, all participants are insured in case of serious AEs.

The original Case Report Form (CRF) and all other forms (including consent forms) will be archived in a locked cabinet at Kyung Hee University Korean Medicine Hospital for 10 years before being destroyed. The trial database is anonymized and securely held using passwords. The datasets generated or analyzed during the study are not publicly available due to privacy concerns. The results of this trial will be published in a peer-reviewed international journal.

## Discussion

To our knowledge, this is the first pilot RCT protocol to evaluate the efficacy and safety of acupuncture treatment for patients with fatigue after COVID-19 and to explore the feasibility of future full-scale RCTs. Although it was a pilot RCT, we designed this trial to meet the rigorous methodological principles of adequate power, randomization, blinding, and inert sham acupuncture intervention.

For the control group, non-penetrating sham needles developed by Park et al. (Dong Bang Acuprime, United Kingdom) will be used (21, 23). This needle comprises a plastic base, two types of tubes, and a disposable needle. The guide tube is to keep the blunt needle vertically standing while other larger tube called a “Park tube” is attached to a silicon base. The Park tube allows the guide tube to move along its interior. Whichever patient is designated, this set of silicon bases, Park tubes, and guide tubes is attached to the acupuncture point. The verum acupuncture needle penetrates the skin inside the sterilized guide tube, whereas the blunt sham acupuncture needle only contacts the skin. As practitioners push the top of the sham acupuncture needle shaft, the blunt tip can transmit the feeling of piercing the skin. This sham acupuncture method has been validated and found to be indistinguishable from verum acupuncture (30). Although there are various methods for sham control such as minimal acupuncture, shallow skin penetration, and non-acupuncture point insertion, these methods are physiologically active and inadequate for comparing the efficacy of acupuncture treatments (31). Furthermore, the study design with sham controls could not blind the patients to the allocation.

In KM real-world practice for fatigue, moxibustion is more commonly used than acupuncture. Moreover, a previous study found that moxibustion was more effective than acupuncture in patients with chronic fatigue syndrome, although both treatments improved fatigue symptoms (32). Therefore, our preliminary study applied moxibustion treatment for fatigue in Long COVID. However, there were a few cases of adverse events (mild blisters at the treatment sites), although all practitioners conducted the same treatment protocol with modulation according to the subjects’ skin condition. In addition, compared to acupuncture treatment, it was difficult to establish an appropriate sham control group. Considering these feasibility aspects from a preliminary study last year, we planned a study protocol to evaluate the efficacy and safety of acupuncture treatment for Long COVID fatigue.

Likewise, as a preliminary pilot RCT, it is possible to find several potential problems before designing future RCT. For example, we might carefully supplement secondary outcome measurements on psychological factors such as anxiety, concentration, cognition, and stress. Furthermore, while the current clinical trials have rapidly accumulated, some treatments (e.g., transcranial direct current stimulation) have demonstrated efficacy on fatigue in post-COVID patients. Therefore, we might consider 3-arm RCT to compare the effectiveness of acupuncture, sham acupuncture and usual care (33).

Our study has several limitations. First, there is the possibility of bias, such as local effect bias and selection bias, since it is a single-center pilot RCT, which would not reflect the diverse characteristics of the study participants. Future full-scale RCTs to identify the exact effect size of acupuncture for fatigue will be conducted at multiple institutions to overcome this limitation. Secondly, the sample size is small. As this is the first pilot RCT on Long COVID fatigue, we could not directly calculate the sample size from the results of relevant previous studies. Therefore, the data from this study will be applied to the sample size calculation in the next full-scale RCT. Third, as the incidence rate continues to decrease after the COVID-19 pandemic has passed a major crisis, the number of patients with COVID-19 is also expected to diminish. It could induce potential challenges in recruiting the target number of subjects. Therefore, we will try to actively promote and recruit by using advertisement on subway and notice board. The last is the non-blinding of the practitioners. Considering the complex characteristics of acupuncture, blinding acupuncture practitioners is impossible. Instead, we will try to blind patients and assessors, and the degree to which the patient is blinded to the group assignment will be evaluated using a blinding index analysis at the end of the trial.

## Conclusion

In conclusion, this pilot study will contribute to clinical evidence on the efficacy and safety of acupuncture treatment for fatigue after COVID-19.

## Ethics statement

The studies involving humans were approved by Institutional Review Board (IRB) of Kyung Hee University Korean Medicine Hospital (KOMCIRB 2023-04-006-001). The studies were conducted in accordance with the local legislation and institutional requirements. The participants provided their written informed consent to participate in this study.

## Author contributions

S-AK: Conceptualization, Investigation, Methodology, Writing – original draft. J-SL: Conceptualization, Investigation, Methodology, Writing – original draft. TK: Investigation, Methodology, Writing – review & editing. T-HK: Methodology, Project administration, Writing – review & editing. SK: Funding acquisition, Methodology, Project administration, Writing – review & editing. JK: Conceptualization, Methodology, Supervision, Writing – review & editing.
