# Supplementary material for: Efficacy and safety of acupuncture treatment for fatigue after COVID-19 infection: study protocol for a pilot randomized sham-controlled trial
Source: Front Neurol. 2023 Nov 15;14:1302793. doi: 10.3389/fneur.2023.1302793 (PMC10684676; doi:10.3389/fneur.2023.1302793)
Supplement: Supplementary file 1 [file Data_Sheet_1.docx]

**Supporting information 1: STandards for Reporting Interventions in Clinical Trials of Acupuncture (STRICTA)**

| Items | Detail |  |
| --- | --- | --- |
| 1. Acupuncture rationale | 1a) Style of acupuncture | Manual acupuncture, Korea |
|  | 1b) Reasoning for treatment provided, based on historical context, literature sources, and/or consensus methods, with references where appropriate | The acupuncture points were selected according to the consensus of board-certified professional KM doctors based on a textbook, literature review, and the points used in our previous prospective case series study. |
|  | 1c) Extent to which treatment was varied | None |
| 2. Details of needling | 2a) Number of needle insertions per subject per session (mean and range where relevant) | Ten acupuncture points were chosen. |
|  | 2b) Names (or location if no standard name) of points used (uni/bilateral) | Ten acupuncture points were chosen: CV12, CV4, bilateral LI4, SP6, ST36, and LI11, in accordance with the WHO Standard Acupuncture Point Locations. |
|  | 2c) Depth of insertion, based on a specified unit of measurement, or on a particular tissue level | a depth of 5-20 mm, depending on the anatomical location of the acupuncture point. |
|  | 2d) Response sought (e.g. de qi or muscle twitch response) | “de qi” |
|  | 2e) Needle stimulation (e.g. manual, electrical) | After insertion, needles will be twisted several times until the participants feel “de qi” sensation and be left for 20 min. |
|  | 2f) Needle retention time | 20-minute needle retention |
|  | 2g) Needle type (diameter, length, and manufacturer or material) | . Sterilized disposable 25 mm × 40 mm acupuncture needles (Dong Bang, South Korea) |
| 3. Treatment regimen | 3a) Number of treatment sessions | 12 sessions |
|  | 3b) Frequency and duration of treatment sessions | all participants will be treated for a total of 12 sessions (three times per week for four weeks) |
| 4. Other components of treatment | 4a) Details of other interventions administered to the acupuncture group (e.g. moxibustion, cupping, herbs, exercises, lifestyle advice) | Infrared therapy will be administered to the abdomen during needle retention. |
|  | 4b) Setting and context of treatment, including instructions to practitioners, and information and explanations to patients | As all pharmacological or CAM interventions for any reasons will be not prohibited during the trial period, it will be recorded narratively at every visits. |
| 5. Practitioner background | 5) Description of participating acupuncturists (qualification or professional affiliation, years in acupuncture practice, other relevant experience) | Acupuncture treatment will be performed by certified practitioners with at least six years of KM education and more than two years of clinical experience. |
| 6. Control or comparator interventions | 6a) Rationale for the control or comparator in the context of the research question, with sources that justify this choice | non-penetrating needles devised by Park et al. (Acuprime, UK) |
|  | 6b) Precise description of the control or comparator. If sham acupuncture or any other type of acupuncture-like control is used, provide details as for Items 1 to 3 above. | For the sham acupuncture treatment group, 25 mm × 45 mm-size non-penetrating needles devised by Park et al. (Acuprime, UK) will be used. These are sterilized disposable blunt needles that only contact the skin without penetration and will be propped by a validated guiding tube called the Park Sham Acupuncture Device (Dong Bang Acuprime, UK) which will be attached to the same acupuncture points as those of the verum acupuncture group. There will be no manipulation of qi, and the needles will be retained for 20 min. |
