# Supplementary figures and images for: Efficacy and safety of acupuncture treatment for fatigue after COVID-19 infection: study protocol for a pilot randomized sham-controlled trial
Source: Front Neurol. 2023 Nov 15;14:1302793. doi: 10.3389/fneur.2023.1302793 (PMC10684676; doi:10.3389/fneur.2023.1302793)

**Supporting information 2. Hospital setting**


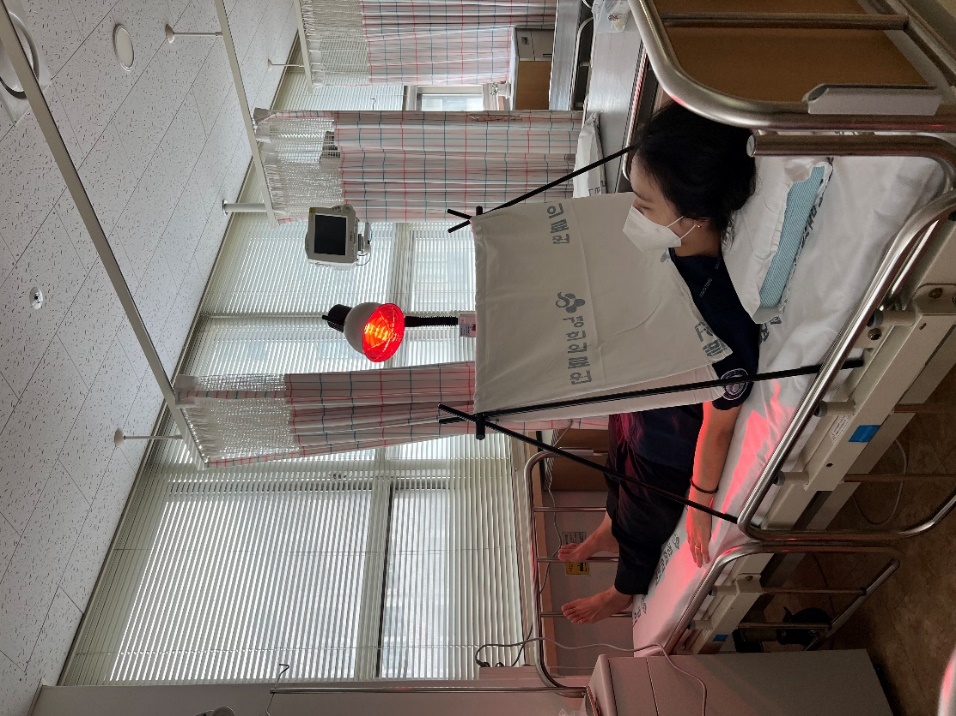


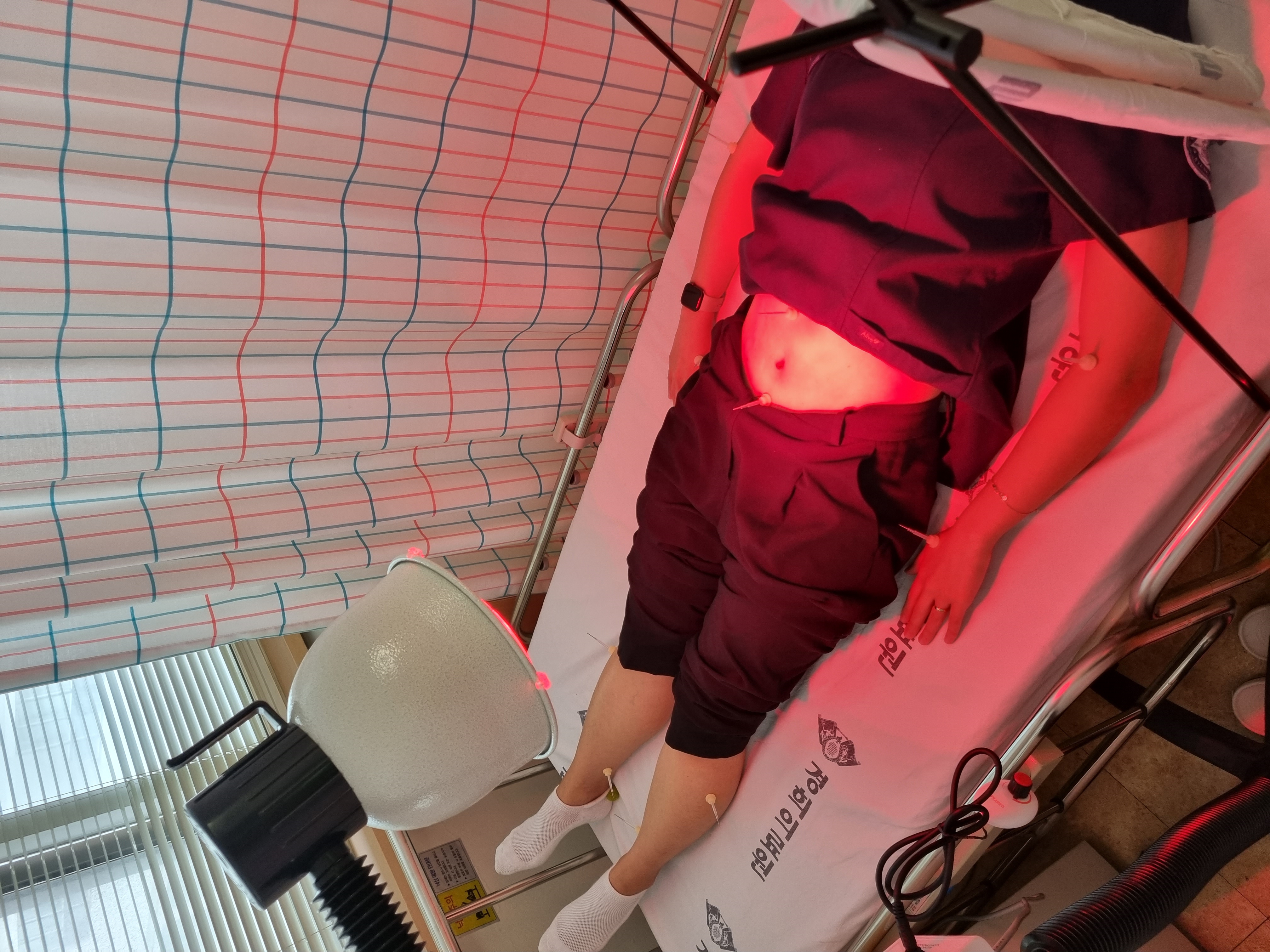


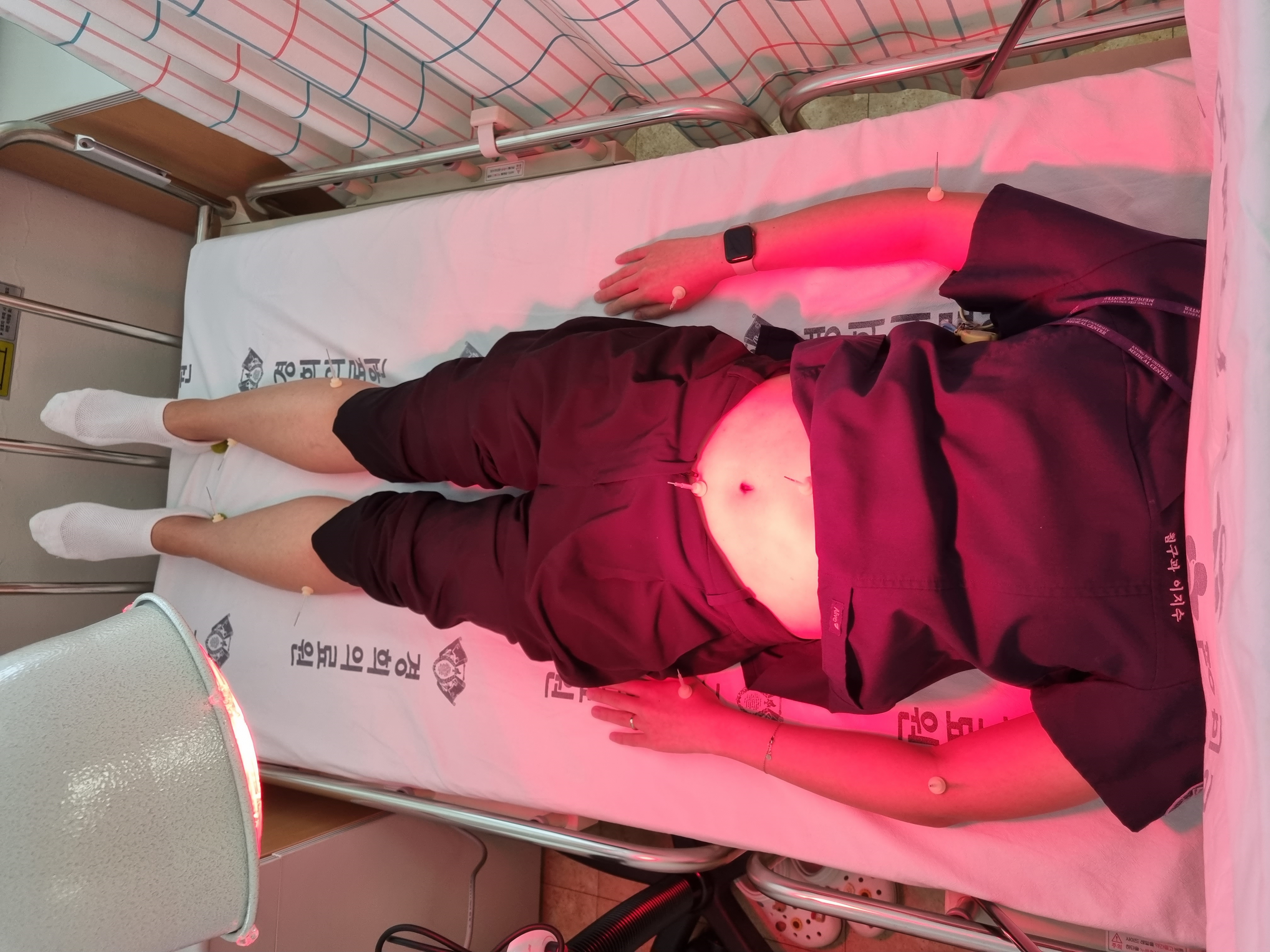

Supplement: Supplementary file 2 [file Data_Sheet_2.docx]
